# The Th1:Th2 Dichotomy of Pregnancy and Preterm Labour

**DOI:** 10.1155/2012/967629

**Published:** 2012-06-07

**Authors:** Lynne Sykes, David A. MacIntyre, Xiao J. Yap, Tiong Ghee Teoh, Phillip R. Bennett

**Affiliations:** ^1^Parturition Research Group, Department of Surgery and Cancer, Institute of Reproduction and Developmental Biology, Imperial College London, London W12 0NN, UK; ^2^St Mary's Hospital, Imperial College Healthcare NHS Trust, London W1 2NY, UK

## Abstract

Pregnancy is a unique immunological state in which a balance of immune tolerance and suppression is needed to protect the fetus without compromising the mother. It has long been established that a bias from the T helper 1 cytokine profile towards the T helper 2 profile contributes towards successful pregnancy maintenance. The majority of publications that report on aberrant Th1:Th2 balance focus on early pregnancy loss and preeclampsia. Over the last few decades, there has been an increased awareness of the role of infection and inflammation in preterm labour, and the search for new biomarkers to predict preterm labour continues. In this paper, we explore the evidence for an aberrant Th1:Th2 profile associated with preterm labour. We also consider the potential for its use in screening women at high risk of preterm labour and for prophylactic therapeutic measures for the prevention of preterm labour and associated neonatal adverse outcomes.

## 1. Introduction

Preterm labour occurs in some 10% of pregnancies [1]. In many developed countries, the rates are rising. Birth before 37 weeks of gestation is thought to account for up to 70% of neonatal deaths, and the extremely high neonatal intensive care costs required to support those who do survive make preterm birth both a social and economic burden. It is now widely acknowledged that the aetiology of preterm labour is multifactorial, and, as such, the underlying cause of preterm labour is often unknown. There is a strong association between preterm labour and infection and inflammation, and research in this field has dramatically increased over the last few decades [2]. However, we still have made little significant progress in the prevention of preterm labour. Evidence of the detrimental direct impact of maternal infection/inflammation on neonatal outcome is emerging, yet we do not fully understand if anti-inflammatory therapeutic agents would provide benefit or harm to the neonate born under conditions of infection/inflammation-induced preterm labour.

The immunology of pregnancy is complex, in that the mother must tolerate the “foreign” fetus, and thus requires a degree of immunosuppression whilst on the other hand needs to maintain immune function to fight off infection. One mechanism which is involved in successful pregnancy maintenance is the proposed switch from the T helper 1 (Th1) cytokine profile to the T helper 2 (Th2) profile. This paper explores the evidence for an imbalance in the Th1:Th2 profile in women at risk of and who are in established preterm labour.

## 2. The Immunology of Pregnancy

**Figure 1:**
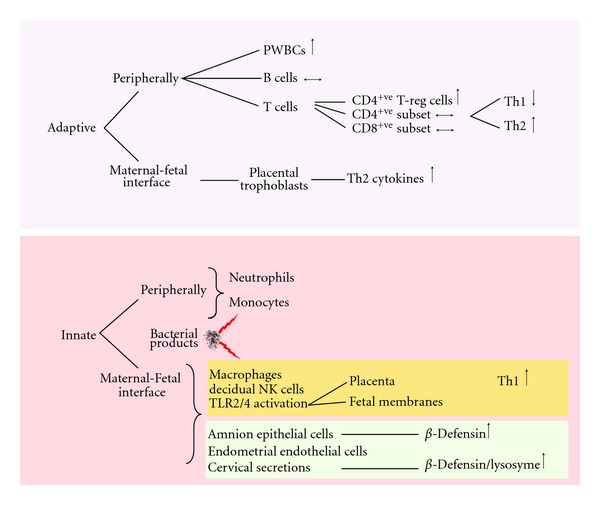
Summary of the adaptive and innate immune system in pregnancy. Mediators of the adaptive and innate immune system work in parallel to facilitate a balance between immune tolerance of the fetus whilst maintaining the ability to mount a response against invading pathogens. PWBC: peripheral white blood cells.

The fetus can be described as a semiallogeneic graft, being a product of two histoincompatible individuals [3, 4]. This poses a challenge to the mother, to both tolerate and accommodate the fetus, which will express paternal antigens, and maintain an ability to reject in case of overwhelming infection [5]. This challenge is undertaken in part by the immune system. The immune system has two main defence systems: the innate and the adaptive. The innate immune response is a nonspecific reaction towards foreign antigens, whereas the adaptive response forms a very specific reaction towards antigens [6]. Although different immune components are involved in these systems, much overlap and cross-talk exist between the two.  Figure 1 summarises the key elements of these systems during pregnancy.

The immune cells that make up the adaptive immune response include B and T lymphocytes. Activation by antigen presenting cells and cytokines leads to cytokine release by T cells in a cell-mediated response, or antibody release by B cells in a humoral response [7]. Although Medawar originally hypothesised that pregnancy represents a time of immune suppression [8], a more complex picture has recently emerged where a change in the ratio and function—rather than a complete suppression—of the maternal leukocytes occurs during pregnancy. For example, there is an increase in the total peripheral white cell count from the early stages of pregnancy with no change in the CD4 and CD8 counts [9]. Within the CD4 positive population, an increase in T regulatory cells is seen in pregnancy [10]. The function of the T cells adapts in pregnancy to favour the T helper 2 cytokine profile, which is more pronounced at the maternal fetal interface [11]. Nonimmune cells, for example, placental trophoblasts also contribute to the Th2 cytokine predominance in pregnancy [12].

The innate immune system provides a less specific response nevertheless is critical for the prevention of microbial invasion. Cellular components include neutrophils, monocytes, and macrophages, which protect against pathogens by phagocytosis. The Toll-like receptors (TLRs) TLR2 and TLR4 are pattern recognition receptors stimulated by Gram-positive and Gram-negative bacteria, respectively [1]. TLRs are expressed on nonimmune cells in the placenta and fetal membranes, which mediate part of the innate immune system at the maternal fetal interface [13]. TLR2 and 4 mutations are associated with an increased risk of preterm birth [14, 15]. During pregnancy, there is tight regulation and considerable cross-talk between the adaptive and the innate adaptive immune system that is responsible for preventing or activating rejection of the conceptus.

## 3. Th1:Th2 Cytokines

T helper 1 and 2 cell subsets originate from undifferentiated Th0 cells under the influence of interferon-gamma (IFN-*γ*) and interleukin-4 (IL-4), respectively. Pregnancy hormones such as progesterone [16], leukaemic inhibitory factor [17], estradiol [18], and prostaglandin D_2_ (PGD_2_) [19] promote the T helper 2 cell profile and are likely to be in part responsible for the Th2 bias associated with pregnancy.

Type 1 CD4^+^ T cells (Th1) produce an array of inflammatory cytokines including IFN-*γ* [20], IL-2 [21], and Tumor necrosis factor-alpha (TNF-*α*) [22] and are the major effectors of phagocyte-mediated host defence, protective against intracellular pathogens [21, 23, 24]. Type 2 CD4^+^ T cells (Th2) produce IL-4, IL-5, IL-13, IL-10 [20], and IL-6. Whilst IL-4 and IL-10 are considered to be anti-inflammatory cytokines [25], IL-6 has proinflammatory properties [26]. Although IL-10 and IL-6 are frequently referred to as Th2 cytokines [27–32], they are both produced by other cell types including Th1 cells, macrophages, and B cells for IL-10 [33, 34], and macrophages, fibroblasts, and B cells for IL-6 [35]. The T helper 2 cytokines are commonly associated with strong antibody responses [36], for example, IL-4 stimulates IgE and IgG_1_ antibody production [37]. However, the Th2 cytokines also serve other functions, for example; IL-5 promotes the growth and differentiation of eosinophils, whereas IL-13 and IL-10 inhibit the activity of macrophages [37]. T helper 2 cell responses are also associated with protection against parasites, since IL-4 mediates IgE production, and IL-5 mediates an eosinophilia, both of which are hallmarks of parasitic infection [38]. It is important to note that, although the Th1 and Th2 responses can be seen as discrete responses, there is considerable cross-talk and overlap between the functions of the T helper cells. For example, the Th1 cytokines can promote the production of complement-fixing antibodies involved in antibody-dependent cell cytotoxicity [39], and thus the dichotomy described may be an oversimplified representation of the complex immune system. Transcriptional regulation of the predominant Th2 cytokine IL-4 is by STAT-6, c-maf, GATA-3, and NFAT [40], whereas Th1 cell cytokine production is transcriptionally regulated by T-bet and STAT-4 [11].

## 4. Th1:Th2 and Pregnancy Maintenance

The hypothesis of Th2 predominance and downregulation of the Th1 response originated from Wegmann and colleagues [41] and was reinforced by evidence from both murine studies and the clinical course of Th2 and Th1 based conditions in pregnancy. IL-2, IFN-*γ*, and TNF-*α* induce miscarriage in mice, which can be reversed by inhibitors of the Th1 cytokines or by administering the anti-inflammatory Th2 cytokine IL-10 [42, 43]. Autoimmune conditions where Th1 is involved in the pathophysiology generally improve in pregnancy (e.g., rheumatoid arthritis [44]), whereas the Th2 autoimmune spectrum tends to worsen (e.g., systemic lupus erythematosus [45]). With a Th2:Th1 bias, the diminished cell-mediated immunity may be responsible for the increased susceptibility in pregnancy of conditions caused by intracellular pathogenesis (e.g., influenza, leprosy, and *Listeria monocytogenes *[46]).

### 4.1. Peripheral Blood

Several techniques are available to establish the function of Th1 and Th2 cells in pregnancy; enzyme-linked immunosorbent assay (ELISA) can be used to measure maternal serum interleukins; peripheral T cells can be isolated and stimulated with a mitogen such as phorbol myristate acetate (PMA) or phytohaemagglutinin (PHA) to measure the cytokine production either by ELISA or flow cytometry during pregnancy compared with nonpregnant controls.

Marzi and colleagues isolated PBMCs, stimulated them with PHA, and measured interleukin secretion by ELISA showing a reduction in IFN-*γ* and IL-2 and an increase in IL-4 and IL-10 in pregnancy compared with nonpregnant controls [47]. In support of this study, Reinhard et al. stimulated cells with PMA and demonstrated by flow cytometry a reduction in intracellular IFN-*γ* and IL-2, and an increase in intracellular IL-4 production in pregnancy compared with nonpregnant controls [48]. *In vivo* confirmation of this bias has since been demonstrated by polymerase chain reaction (PCR) reflecting decreasing messenger ribonucleic acid (mRNA) of IFN-*γ* through pregnancy and a concurrent increase in IL-4 mRNA which peaks in the 7th month compared with nonpregnant controls [49]. However, not all studies support the Th1 to Th2 bias. Shimaoka et al. reported a reduction in PMA-stimulated IL-4 during pregnancy [50], while Matthiesen and colleagues presented data suggesting an increase in both IL-4 and IFN-*γ* secreting cells in pregnancy compared with nonpregnant controls [51, 52]. Such discrepancies may be due to characterisation of cytokine profiles in either isolated cell populations or whole blood, the latter arguably being a more biologically relevant system.

### 4.2. Maternal Fetal Interface and Nonimmune Cells

While much research has been dedicated toward circulating cytokines in pregnancy, local cytokine production at the maternal interface may be of greater significance than measurements obtained in the peripheral blood [23]. IL-4, IL-10, and macrophage colony-stimulating factor (m-CSF) production by T cells at the maternal fetal interface is associated with successful pregnancy [23]. Trophoblast, decidua, and amnion all contribute to the Th2 cytokine environment by production of IL-13 [53], IL-10 [54], IL-4 and IL-6 [55, 56]. Coculture of trophoblasts and T cells results in an increase in the transcription factors GATA-3 and STAT-6 (which regulate Th2 cytokine production), and a reduction in the Th1 transcription factor STAT-4 and subsequently decreased production of IFN-*γ* and TNF-*α* [57]. The placenta also synthesises PGD_2_, which may act as a chemoattractant of Th2 cells to the maternal fetal interface via the classic Th2 receptor CRTH2 (chemoattractant receptor-homologous molecule expressed on Th2 cells) [28]. CRTH2^+^ cells are reduced at the maternal fetal interface of women suffering from recurrent loss compared with women undergoing elective termination [58].

Local production of IL-4 and IL-10 inhibits the function of both Th1 cells and macrophages, which serves to prevent fetal allograft rejection [59]. Other anti-inflammatory effects of these interleukins result in inhibition of the Th1 cytokine TNF-*α* [60], and TNF-*α*-induced cyclo-oxygenase-2 (COX-2), and/or PGE_2_ synthesis in amnion-derived wish cells. Similar effects are observed in decidual and placental cells *in vitro* [61–64], which is thought to inhibit the onset of labour. Consistent with such a role, decidual CD4 positive cells from women undergoing unexplained recurrent pregnancy loss typically exhibit reduced IL-4 and IL-10 production [65].

## 5. Th1:Th2 Cytokines in Labour

### 5.1. Peripheral Blood

The Th2 cytokine predominance which exists during pregnancy has been shown to return to nonpregnant Th1:Th2 ratios by 4 weeks postpartum [66]. Labour is often seen as a proinflammatory state marking the end of the pregnancy, and thus it is plausible that labour is associated with a reversal in the bias back towards Th1 rather than Th2. Rather, Kuwajima and colleagues have shown that the Th2:Th1 ratio remained constant in favour of Th2 through pregnancy and labour, with a reversal back to nonpregnant parameters at 7 days postpartum [67]. However, this finding is somewhat contrary to an earlier report indicating that serum IL-4 levels measured by ELISA in women through pregnancy and at different stages of labour were reduced in the later part of labour and by day 1 postpartum in both normotensive and preeclamptic women [68]. In this study, the Th1 proinflammatory cytokine TNF-*α* peaked in early labour consistent with labour being a proinflammatory state. Consistent with this, an increase in IFN-*γ* and IL-1*β* in women in active labour has also been reported [69].

### 5.2. Maternal Fetal Interface and Nonimmune Cells

There is substantial evidence that the Th1 cytokines play a role in the initiation of labour at term [22]. The importance of local rather than peripheral production of the cytokines is highlighted by their direct input into the biochemical pathways involved in parturition. Fetal membranes [70, 71] and myometrium [72] produce IL-1*β* at term, a potent inducer of NF-*κ*B [73]. This transcription factor regulates the expression of numerous labour-associated genes including COX-2, the oxytocin receptor, IL-8, and matrix metalloproteinase-9 (MMP-9) [74]. TNF-*α* and IL-1*β* are both increased in amnion, amniotic fluid, and decidua at term [75] and can induce PGE_2_ production in amniocytes and decidual cells *in vitro *[76, 77]. Despite the proinflammatory nature of the Th1 cytokines they are required for successful pregnancy contributing to the physiology of term labour.

## 6. Th1:Th2 Cytokines in Infection

Activation of the Th1 cytokines occurs as a specific response to infection caused by intracellular bacteria, parasites, and viruses [78]. The necessary proinflammatory type 1 response elicited by infection, along with the action of the activated T cells, drives local and systemic cytokine production that, if left unchecked, can be harmful to the host [78]. In some situations, the Th1 response is balanced by the production of Th2 cytokines, particularly IL-4 and IL-10 [79–82]. In the early stages of infection, IL-12 is produced by macrophages and dendritic cells [83, 84], which lead to polarisation from Th0 to Th1 type cells [24]. IFN-*γ* enhances Th1 development by upregulating the IL-12 receptor and inhibiting the growth of Th2 cells [85]. IFN-*γ* also primes macrophages to begin phagocytosis and to stimulate the release of interleukin-1 [86].

While the Th1 cytokine response may be suppressed by both the maternal and fetal immune system during pregnancy [87], it still maintains the capacity to mount a defensive response in the context of infection. For example, cord blood mononuclear cells cultured with lipopolysaccharide (LPS) *in vitro* show an increased production of IFN-*γ* concurrent with reduced IL-4 secretion [88]. Similarly, neonates exposed to intrauterine infection have an increased percentage of IFN-*γ*-producing cells, with some neonates also showing an increase in IL-4-producing cells [89]. In response to LPS amnion, chorion, deciduas, and placenta also release proinflammatory cytokines [64, 90, 91].

## 7. Th1:Th2 Cytokines in Preterm Labour

Approximately, 30% of preterm births are associated with infection [92], with a higher rate of 80–85% in early preterm birth (<28 weeks) [93]. Immune and nonimmune cells contribute to a cytokine-rich environment in the presence of infection and inflammation. Proinflammatory cytokines such as TNF-*α* and IL-1*β* ultimately result in the production of prostaglandins and MMPs [86], via NF-*κ*B. This triggers a cascade of prolabour events including uterine contractility and fetal membrane rupture, and if this cascade is activated early in pregnancy, preterm labour can ensue.

### 7.1. Peripheral Blood

As discussed above, the peripheral response may not be as potent as the local Th1:Th2 response and may instead reflect a more significant inflammatory response at the fetal placental compartment. A large case control study of 101,042 Danish women showed that an elevated mid pregnancy IFN-*γ* plasma level was associated with moderate and late spontaneous preterm delivery, whereas no increased risk was seen with elevated TNF-*α* or IL-2 [94]. However, a study comparing women in active preterm labour and no labour looked at mitogen-stimulated production of IFN-*γ* and the Th2 cytokines IL-4, IL-10, and IL-13 and showed no difference in median cytokine production in the supernatant *in vitro* [86]. The differing results between these studies could be explained by the fact that the *in vitro *cells lack the presence of other cells of the immune system and thus lack the ability to reflect the complexity of the immune system as a whole. This same study did however show a higher IL-12 and lower IL-4 in cervical secretions of women in preterm labour, reflecting the localised Th1:Th2 dichotomy. Bahar and colleagues did not demonstrate any difference in serum TNF-*α* or IFN-*γ* in women with preterm labour compared to term labour or matched controls not in labour [95]. However, those women in the preterm labour group received indomethacin, an anti-inflammatory COX-2 inhibitor, which could have dampened a typical proinflammatory response. Serum taken from women with preterm prelabour rupture of membranes (pPROM) compared to women who delivered at term exhibit a higher concentration of IFN-*γ*. Levels of IL-4 and IL-5 were undetectable in both groups [96]. In a study of 30 women in preterm labour, mitogen- and antigen-stimulated PBMCs showed a higher production of the pro-inflammatory cytokines IFN-*γ* and IL-2, along with an altered Th1:Th2 ratio favouring a Th1 response compared with controls who delivered at term [97]. Taken together, these results suggest that, rather than a decrease in the Th2 response, preterm labour most likely represents an activation of the Th1 response. Thus, future development of therapeutic targets would likely be more effective if directed towards the modulation of the Th1 cytokines.

The Th1:Th2 dichotomy likely represents an oversimplification of the complexity of the cross-talk between the Th1 and Th2 cytokines. The ratio of Th1:Th2 is likely to be of more physiological importance than the actual concentrations produced. In support of such a notion, women in threatened preterm labour with high serum levels of IL-12 (which induces a Th1 cytokine response) and no change in serum IL-18 (which can induce both Th1 and Th2 response) do not show significant associations with preterm labour. However, women with high IL-12 levels and low IL-18 and thus a high IL-12 : IL-18 ratio increasing the Th1 predominance are associated with a twofold risk of preterm labour when presenting with threatened preterm labour [98].

### 7.2. Maternal Fetal Interface and Nonimmune Cells

The inflammatory response at the maternal fetal interface more likely reflects the true importance of the Th1:Th2 dichotomy and the aberrant profile in preterm labour. A recent meta-analysis concluded that proinflammatory cytokines at the maternal fetal interface play a role in the events leading to spontaneous preterm labour, while systemic inflammation does not appear to be present in asymptomatic women early on in pregnancy who then go on to deliver preterm [99]. This is consistent with a more local intrauterine inflammatory response syndrome, where no organisms are identified. Understanding the pathophysiology at the maternal interface is essential for developing new therapies for the prevention of inflammation-induced preterm labour, although using such local changes for the prediction is challenging because of lack of access to the maternal fetal interface.

Placentas from women with pPROM and preterm delivery have higher Th1, inducing cytokines [100], and placentas from women following preterm delivery compared with term delivery show a bias towards the Th1 profile with significantly higher levels of IFN-*γ* and IL-2 as well as the Th1-inducing cytokine IL-12 [100]. Moreover, term placentas exhibit comparatively higher levels of the Th2 cytokines, IL-4, and IL-10, compared with the preterm placentas.

TNF-*α* is increased in choriodecidual tissues [71] and amniotic fluid [101] in preterm labour. TNF-*α* is known to stimulate PG production through the TNF receptor 2, leading to uterine contractions likely via activation of NF-*κ*B, but is also likely to contribute to MMP-9 production leading to PROM via activation of its receptor TNF Receptor 1 (TNFR1) [102]. Interestingly, samples of myometrium collected women in preterm labour and samples collected preterm before labour express comparable mRNA levels of TNF-*α*. However, mRNA levels of the receptors, TNF R1 A and B, are increased in preterm labour and term labour compared with nonlabour controls [103] suggesting a receptor-mediated increase in sensitivity to TNF-*α*.

Although placental, amnion, and choriodecidual cells secrete proinflammatory cytokines, cytokine levels in tissues from preterm deliveries (with and without intrauterine infection) correlate with the extent of leukocyte infiltration in fetal membranes [75]. In the presence of infection, the primary cellular source of cytokine production in fetal membranes is likely to be infiltrating leukocytes rather than amniocytes or choriodecidual cells. [75].

### 7.3. Polymorphisms of the Th1 and Th2 Cytokines

Studying genetic polymorphisms of the Th1 and Th2 cytokines could provide a novel screening method for determining women at high risk of preterm labour. Polymorphisms giving rise to functional alterations can also provide information on the importance of the interleukins in preterm labour. There has yet to be any promising genetic polymorphisms identified in the Th1:Th2 cytokines for the prediction of preterm labour, the work conducted warrants consideration (see Table 1).

## 8. Non-Th1:Th2 Interleukins

### 8.1. IL-8

Interleukin 8 is a chemokine produced by many immune cells but primarily macrophages and monocytes [116]. Its production is stimulated by LPS, TNF, and IL-1 [117] and, in the context of pregnancy, is thought to attract leukocytes to the gestational tissues and the cervix at the onset of term and preterm labour. IL-8 mRNA expression has been reported to be increased more than 50-fold in preterm labour and more than 1000-fold in preterm labour with evidence of chorioamnionitis in amnion and choriodecidua [118]. A number of studies have also identified increases of IL-8 in the myometrium and cervix with the onset of labour [119, 120]. Placental IL-8 is also higher in preterm deliveries compared with term deliveries [71].

### 8.2. IL-6

Although IL-6 is produced by Th2 cells, it is a proinflammatory cytokine and a major mediator of host response to inflammation and infection [121]. IL-6 levels are moderately increased in placenta, significantly increased in amnion and choriodecidua in women with preterm delivery compared with term delivery [71]. IL-6 appears to be among the most sensitive and specific indicators of infection-associated preterm labour [122, 123]. The presence of an increase in IL-6 in amniotic fluid and cervicovaginal fluid is an independent risk factor for preterm labour and neonatal morbidity [124] including cerebral palsy [125] and bronchopulmonary dysplasia [126].

## 9. Therapeutic Modulation of Th1 and Th2 Profile

Various therapeutic strategies have been proposed to prevent preterm labour, with the primary objectives of (1) delaying delivery to increase gestation at delivery and (2) to improve neonatal condition at birth [127]. Currently, many of the strategies adopted for the prevention of preterm labour involve targeting the proposed pathways and events that result in uterine contractions and cervical shortening and dilation rather than targeting immune activation. As described here, an aberrant proinflammatory profile exists in both term and preterm labour, which is associated with neonatal morbidity. The limitation of tocolytics is the inability to counteract the exposure of the fetus to proinflammatory cytokines, which lead to the fetal inflammatory response syndrome. This may in fact worsen neonatal outcome by prolonging the exposure of the fetus to a hostile environment. There is mounting evidence that periventricular leukomalacia and cerebral palsy are associated with fetal exposure to intra-amniotic inflammation and the development of fetal inflammatory response syndrome [128]. Thus, a strategy for targeting immune activation through the modulation of the Th1:Th2 bias may be beneficial for both the prevention of preterm labour as well as the reduction of neurological insult to the fetus.

### 9.1. Progesterone

There have been several studies indicating a positive response to progesterone treatment for the prevention of preterm labour in specific patient populations [129–131]. The strongest evidence for improvement in neonatal outcomes comes from the most recent multicentre randomised controlled trial which showed a 45% reduction in preterm labour (<33 weeks) and a 60% reduction in respiratory distress syndrome at <33 weeks using 90 mg of vaginal progesterone in women with a short cervix of 10–20 mm [132]. The mechanism by which progesterone contributes to pregnancy maintenance has traditionally been attributed to maintenance of uterine quiescence by increasing cyclic AMP (cAMP) and a reduction in intracellular calcium thus reducing contractility [133]. Moreover, progesterone appears to inhibit the phosphorylation of myosin, a critical step in the activation of the myometrial contractile machinery required for labour onset [134, 135].

Progesterone also has immunomodulatory effects on the Th1:Th2 bias. Progesterone is able to suppress Th1 differentiation and enhance Th2 differentiation in peripheral blood mononuclear cells *in vitro* [136]. A more potent and orally bioavailable progestogen, dydrogesterone (6-dehydro-9*β*,10*α*-progesterone) upregulates IL-4 and downregulates IFN-*γ* in PHA-stimulated PBMCs more significantly than progesterone *in vitro* [137]. There is also *in vivo* evidence of an anti-inflammatory effect of prolonged administration of vaginal progesterone. In a study of pregnant women receiving either progesterone or placebo from 24 to 34 weeks, peripheral blood leukocytes were collected before and after treatment [138]. mRNAs of the proinflammatory cytokines IL-1*β* and IL-8 were reduced with progesterone treatment, whereas the anti-inflammatory IL-10 was increased. A multicentre placebo controlled trial (OPPTIMUM, https://www.opptimum.org.uk/: ISRCTN 14568373) powered on neonatal outcome will provide us with evidence of any potential beneficial effect of vaginal progesterone on neonates born preterm.

### 9.2. NF-*κ*B Inhibitors

Inhibition of NF-*κ*B activation is another attractive strategy to prevent preterm labour as NF-*κ*B activation is central to the activation of labour-associated genes in labour [139]. NF-*κ*B activation also leads to a proinflammatory response in various cytokines including IFN-*γ* [140], IL-1*β* [74], TNF-*α*, and IL-8 [141]. *Ex vivo* studies with the anti-inflammatory sulfasalazine suppress LPS-induced IL-6 and TNF-*α* production in fetal membranes via inhibition of translocation of p65 to the nucleus [142]. The reported clinical safety profile of sulfasalazine has been variable [143–145], however, if used in pregnancy is often supplemented with folate. The anti-inflammatory characteristics of the cyclopentenone PG, 15-deoxy-Δ^12,14^-prostaglandin J_2_ (15dPGJ_2_) appears to be derived from its ability to inhibit NF-*κ*B activation in human amnion and myometrial cell culture [146]. We have also shown that 15dPGJ_2_ inhibits activation of NF-*κ*B in human peripheral blood mononuclear cells and reduces the percentage of cells producing the proinflammatory cytokines, IFN-*γ* and TNF-*α*, [147]. Work conducted in our laboratory has also shown that 15dPGJ_2_ is able to delay labour and provide neuroprotection by reducing pup mortality from 75% to 5% in a murine model of inflammation induced preterm labour [148].

## 10. Conclusion

There has been extensive interest in the Th1:Th2 dichotomy for the maintenance of successful pregnancy. A trend towards the Th2 cytokine profile and a suppression of the Th1 cytokine profile appears to exist both in the peripheral blood but more significantly at the maternal fetal interface. Activation of the proinflammatory Th1 profile—rather than suppression of the Th2 profile—is apparent in preterm labour and thus should be considered as the logical target for immunomodulating therapies for the prevention of preterm labour and improving neonatal outcome.

## Figures and Tables

**Table 1:** Cytokine polymorphism associations with preterm labour (PTL).

Gene	Polymorphism	Th1/Th2	Function	Reference
IFN-*γ*	+874A>T	Th1	Classic Th1 cytokine. Proinflammatory. No clear association between IFN-*γ* polymorphisms and PTL	[104, 105]
TNF-*α*	−308G>A	Th1	Regulatory role in PG synthesis elevated at maternal fetal interface controversial link between PTL and TNF-*α* polymorphisms	Refute association [104, 106, 107] Support association [108, 109]
IL-4	−590	Th2	Classic Th2 cytokine. The IL-4 590 C/C genotype is associated with preterm birth but unclear. IL-4-590 SNP has been associated with both low and high IL-4 expression. Link also exists between IL-4 promoter polymorphisms and preterm birth in multiple pregnancies; however, polymorphism actually associated with increased IL-4	[110, 111]
IL-10	−1082G>A −819C>T −592C>A	Th2	Anti-inflammatory Th2 cytokine inhibits production of cytokines, chemokines, and prostaglandins in LPS stimulated amnion, choriodecidual, and placental explants [112–114]. However, no clear association between IL-10 polymorphisms and PTL or adverse neonatal outcome	[104–106, 115].
